# Development and validation of a prediction model for activities of daily living dysfunction among stroke survivors: insights from the CHARLS cohort

**DOI:** 10.1186/s12984-026-02063-x

**Published:** 2026-06-21

**Authors:** Hongying Ren, Wen Wang, Li Tao, Xiaoshu Song, Bin Bai, Min Wang, Xiaoyan Li

**Affiliations:** Department of Rehabilitation Medicine, Beijing Anzhen Nanchong Hospital of Capital Medical University & Nanchong Central Hospital, 97 South Renmin Road, Shunqing District, Nanchong, 637000 Sichuan China

**Keywords:** Stroke, Activities of daily living, Prediction model, Rehabilitation, CHARLS

## Abstract

**Background:**

Activities of daily living (ADL) dysfunction is prevalent in stroke survivors and places a significant burden on both patients and healthcare systems. Improved identification of individuals with ADL dysfunction may facilitate more targeted rehabilitation strategies.

**Methods:**

The China Health and Retirement Longitudinal Study (CHARLS) provided the data. A training set (*n* = 906) and a validation set (*n* = 389) were randomly selected from a total of 1,295 stroke survivors. Least absolute shrinkage and selection operator (LASSO) regression and multivariable logistic regression were used to select predictors and develop a prediction model, which was visualized using a nomogram. SHapley Additive exPlanations (SHAP) were applied for model interpretation. The area under the receiver operating characteristic curve (AUC), calibration analysis, and decision curve analysis (DCA) were used to evaluate the model’s performance.

**Results:**

Ten predictors were identified, including CES-D scores, age, sleep time duration, drinking, lung disease, social contact, falls, hypertension, arthritis, and sex. SHAP analysis identified CES-D scores as the most influential predictors. The model demonstrated acceptable discriminative ability in both the training set (AUC: 0.76, 95% CI: 0.73–0.79) and validation set (AUC: 0.76, 95% CI: 0.72–0.81). Calibration was satisfactory in both the training and validation sets (Hosmer–Lemeshow test, *P* = 0.16 and *P* = 0.99, respectively). Positive clinical usefulness was suggested by DCA analysis.

**Conclusions:**

The model demonstrated acceptable predictive performance and may assist in identifying individuals with prevalent ADL dysfunction. Further external validation is required before broader clinical application.

**Supplementary Information:**

The online version contains supplementary material available at https://doi.org/10.1186/s12984-026-02063-x.

## Introduction

Globally, stroke is a leading cause of long-term acquired disability worldwide, impacting approximately one in four adults, with up to half of survivors left chronically disabled [1, 2]. Although advances in acute management have improved survival rates, a considerable proportion of patients continue to experience post-stroke motor, sensory, speech, cognitive, and other impairments [3]. Stroke survivors frequently report difficulties in performing activities of daily living (ADL), decreased quality of life, and limited community participation [4]. Nearly 50% of stroke patients show reduced physical function three months after the stroke, and approximately 40% remain functionally dependent in instrumental activities of daily living (IADL) at next three years [5, 6]. Limitations in ADL not only reduce independence and quality of life but also raise the requirement for long-term care and rehabilitation services. Therefore, stroke-related disabilities impose a profound physical, psychological, and socioeconomic burden on survivors, their families, and the healthcare system at large [7].

It is commonly known that stroke survivors have a high prevalence of activity limits, with estimates indicating that between 25% and 74% require a certain degree of assistance from caregivers for performing ADL [8]. Effective solutions are needed to improve long-term results for stroke survivors, driving an increasing demand for rehabilitation services [9]. Post-acute rehabilitation plays a vital part in encouraging functional recovery and ensuring access to appropriate care [10, 11]. However, given the constraints on healthcare resources, optimizing assessment and treatment strategies is essential to enhance functional outcomes while minimizing the long-term costs associated with residual disability [12, 13]. In this context, accurate risk assessment is essential for clinical decision-making in rehabilitation, prompting further research into predictors of ADL dysfunction to optimize functional outcomes after stroke [14].

Accurate prediction of ADL dysfunction plays a key role in optimizing early stroke management, as it supports realistic goal setting, informs discharge planning, and provides reliable guidance for patients and their caregivers [15]. Recovery and transitions between dependence and independence in ADL may continue for up to 12 months after stroke [16, 17], reflecting considerable heterogeneity among patients [18]. Individualized risk assessment strategies can significantly enhance clinical decision-making in rehabilitation by facilitating the development of tailored intervention plans [19]. However, several existing prediction models rely on specialized rehabilitation assessments, such as the Barthel Index, Functional Independence Measure, Trunk Impairment Scale, and other detailed functional evaluations [15, 20, 21], which may constrain their generalizability and practical use in routine rehabilitation care.

The study was reported in accordance with the Transparent Reporting of a multivariable prediction model for Individual Prognosis Or Diagnosis (TRIPOD) statement [22]. Utilizing data from a large, nationally representative cohort, this study sought to identify key factors associated with ADL dysfunction among stroke survivors and to develop and internally validate a diagnostic prediction model for identifying prevalent ADL dysfunction. This model may provide a practical tool for the assessment of ADL dysfunction and support individualized rehabilitation evaluation in stroke survivors.

## Methods

### Study population

**Fig. 1:**
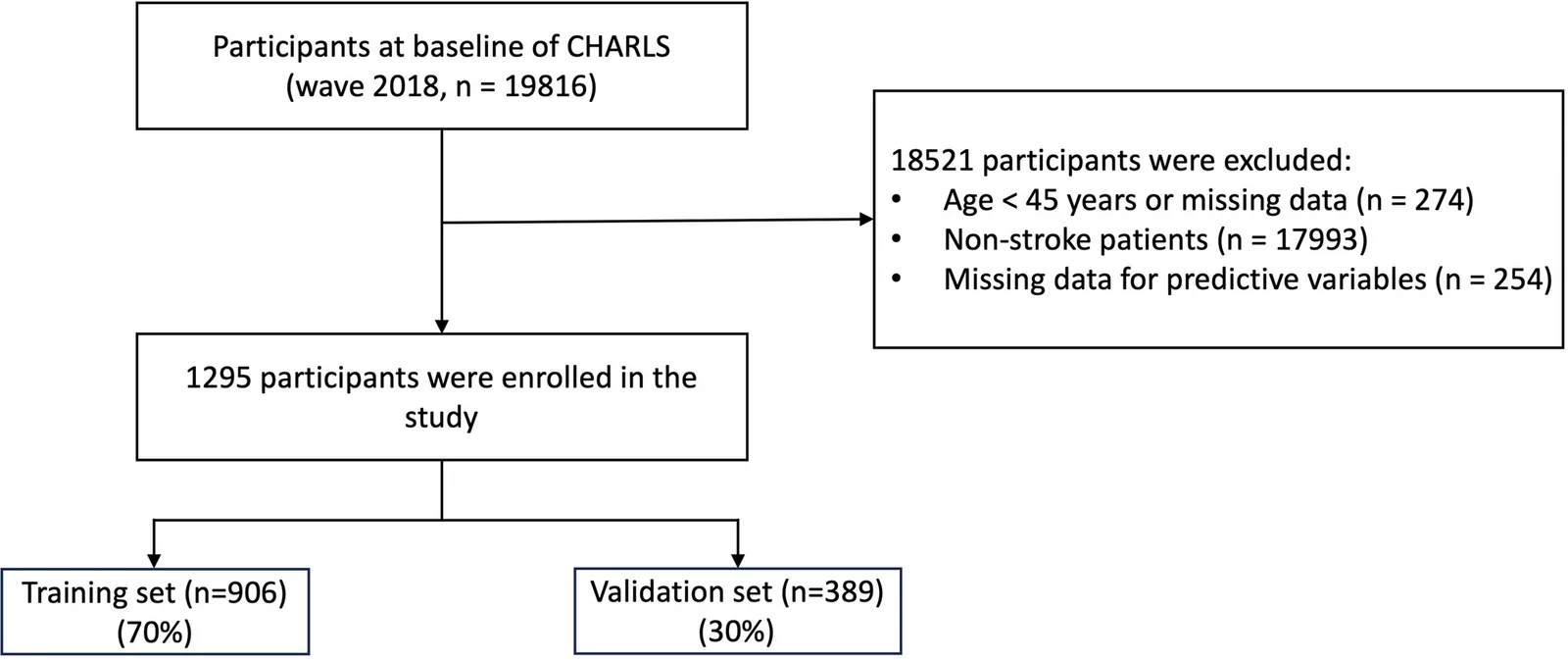
Study flowchart of participant selection process from the CHARLS 2018 dataset

Data were obtained from the China Health and Retirement Longitudinal Study (CHARLS), wave 2018, which initially included 19,816 participants. Individuals aged < 45 years or with missing age information (*n* = 274) were excluded. Participants without a self-reported history of stroke were further excluded (*n* = 17,993). In addition, individuals with missing data for predictor variables were excluded (*n* = 254). Following the application of these standards, 1,295 stroke patients were incorporated into the final analysis. The participant selection process is summarized in Fig. 1. The eligible individuals were then split into a training set and a validation set at random in a 7:3 ratio using the sample function in R software with a fixed random seed of 1234. The model was developed using the training set (*n* = 906) and internally validated using the validation set (*n* = 389).

### Outcome definition

ADL dysfunction was determined using validated instruments for basic activities of daily living (BADL) and IADL [23]. BADL evaluation utilized the Katz ADL scale, covering six self-care activities: eating, bathing, dressing, transferring, toileting, and continence [24]. IADL assessment employed the Lawton IADL scale, which examines five tasks associated with independent living: shopping, cooking, housework, managing finances, and medication management [25]. Each item required participants to report the level of difficulty experienced when performing the activity, with response options including “no difficulty”, “have difficulty but can still do it”, “have difficulty and need help”, and “cannot do it”. Following established practice in prior studies, responses were dichotomized into no difficulty (score = 0) and any difficulty (score = 1) [26, 27]. The range of BADL and IADL scores was 0–6 and 0–5, with higher values corresponding to greater limitations in daily functioning. Consistent with previous CHARLS-based studies, participants reporting difficulty in at least one BADL or IADL item (cumulative score ≥ 1) were classified as having ADL dysfunction, whereas those reporting no difficulty across all items were classified as free of ADL dysfunction [28].

### Candidate predictors

Candidate predictors were selected founded on clinical relevance and availability in the dataset, covering sociodemographic characteristics, lifestyle factors, and clinical conditions. Several established predictors of post-stroke functional outcomes, such as stroke severity, lesion characteristics, time since stroke onset, and acute treatment-related factors, were not available in CHARLS and therefore could not be included in the analysis. Sociodemographic variables comprised age, sex, residence, marital status, educational level, family size, and number of healthy children. Lifestyle factors included sleep time duration (continuous and categorical), smoking status, drinking status, daytime napping, and social contact. Sleep time duration was entered into further analyses as a continuous variable. Clinical variables included history of falls, hip fracture, diabetes, hypertension, heart disease, mental disease, arthritis, dyslipidemia, lung disease, and kidney disease. Pain-related variables were also included, comprising the presence of pain and the number of pain sites. Depressive symptoms were assessed using the 10-item Center for Epidemiologic Studies Depression Scale (CES-D), a validated instrument widely used in CHARLS and Chinese populations. Total scores range from 0 to 30, with higher scores indicating more severe depressive symptoms [29, 30]. Depression was described as a CES-D score of ≥ 10 [31], and was analyzed both as a continuous variable (CES-D score) and a categorical variable (depression status).

### Statistical analysis

For data description, continuous measures are reported as either means (± SD) or medians with interquartile ranges (IQR), while categorical data are summarized using frequencies and proportions. Depending on the distribution, differences between groups were assessed via the independent t-test or Mann–Whitney U test, whereas the chi-square test was employed for categorical comparisons. The distribution of missing data is presented in Table S1. Missing values were handled using multiple imputation, with predictive mean matching applied to continuous variables and logistic regression used for categorical variables. As this study was based on a secondary analysis of an existing database, a formal prospective sample size calculation was not performed. Sample size adequacy was assessed using the events-per-variable (EPV) criterion. With 498 participants experiencing ADL dysfunction and 24 candidate predictors considered for model development, the EPV was 20.75, exceeding the recommended minimum threshold of 10 events per variable [32].

In the training set, least absolute shrinkage and selection operator (LASSO) regression was performed for feature selection using the glmnet package in R, with the mixing parameter α fixed at 1 [33]. The optimal penalty parameter (λ) was determined by ten-fold cross-validation based on the minimum cross-validation error (lambda.min), and variables with non-zero coefficients were retained. Variables with non-zero coefficients identified by LASSO regression were simultaneously entered into a multivariable logistic regression model without further stepwise selection. The model was fitted using maximum likelihood estimation to estimate adjusted odds ratios (ORs) and corresponding 95% confidence intervals (CIs). A nomogram was constructed based on the regression coefficients of the final multivariable logistic regression model, with point allocations proportional to the contribution of each predictor. The complete model coefficients are provided in Table S2. Shapley Additive exPlanations (SHAP) analysis was performed using the KernelSHAP method to quantify predictor contributions and improve model interpretability. Variable importance was visualized using summary, global importance, and waterfall plots [34].

Receiver Operating Characteristic (ROC) curve analysis was performed to measure the model’s discriminative performance. The model’s capacity to differentiate between patients with and without ADL dysfunction was evaluated using the area under the curve (AUC) and 95% CIs. As a sensitivity analysis, model performance was further evaluated using complete-case data. In addition to the 70/30 training-validation split, 10-fold cross-validation was also conducted as a sensitivity analysis to further assess model stability and internal validity [35]. Calibration was assessed using calibration plots and the Hosmer–Lemeshow goodness-of-fit test. Given the recognized limitations of the Hosmer–Lemeshow test, calibration results were interpreted alongside graphical assessment of agreement between predicted and observed outcomes [36]. Decision Curve Analysis (DCA) was performed to evaluate the clinical usefulness of the prediction model across a range of threshold probabilities by comparing its net benefit with the treat-all and treat-none strategies [37, 38]. Over a range of clinically relevant threshold probabilities, the model’s net benefit was computed and compared to two default scenarios: “treat-all” (assuming all patients develop dysfunction) and “treat-none” (assuming no patients develop dysfunction).

R software (version 4.2.2) was used for all statistical studies. Statistical significance was defined as a two-sided *P* < 0.05.

## Result

### Baseline characteristics

Table 1 displays the baseline characteristics of the training set participants. Among the 906 stroke patients, 498 (54.97%) had ADL dysfunction and 408 (45.03%) did not. Participants with ADL dysfunction were older than those without ADL dysfunction (67.49 ± 8.57 vs. 64.43 ± 8.72, *P* < 0.001) and had higher CES-D scores (12.00 [7.00, 18.00] vs. 7.00 [4.00, 12.00], *P* < 0.001). A greater proportion of ADL dysfunction was observed among females, rural residents, and individuals with lower educational levels (all *P* < 0.05). Regarding lifestyle factors, participants with ADL dysfunction were less likely to smoke or drink, had shorter or longer sleep durations, and reported less social contact (all *P* < 0.05). In terms of clinical characteristics, ADL dysfunction was linked to a higher frequency of falls, hip fractures, depression, multisite pain, hypertension, heart disease, mental disease, arthritis, kidney disease, and lung disease (all *P* < 0.05). There were no significant differences found between the training and validation sets’ baseline characteristics (Table S3), indicating adequate homogeneity between the two datasets.

**Table 1:** Baseline characteristics of stroke patients with and without ADL dysfunction

Variables	Non-ADL dysfunction (*n* = 408)	ADL dysfunction (*n* = 498)	*P*
Age, Mean ± SD	64.43 ± 8.72	67.49 ± 8.57	< 0.001
Family size, M (Q₁, Q₃)	2.00 (2.00, 3.00)	2.00 (2.00, 3.00)	0.666
Number of healthy children, M (Q₁, Q₃)	2.00 (2.00, 3.00)	3.00 (2.00, 4.00)	< 0.001
CES-D scores, M (Q₁, Q₃)	7.00 (4.00, 12.00)	12.00 (7.00, 18.00)	< 0.001
Sleep time duration, M (Q₁, Q₃)	6.00 (5.00, 7.00)	6.00 (4.00, 8.00)	0.175
Daytime napping, M (Q₁, Q₃)	30.00 (0.00, 60.00)	30.00 (0.00, 60.00)	0.414
Age, n (%)			
< 65	206 (50.49)	171 (34.34)	< 0.001
≥ 65	202 (49.51)	327 (65.66)	
Sex, n (%)			
Female	169 (41.42)	279 (56.02)	< 0.001
Male	239 (58.58)	219 (43.98)	
Residence, n (%)			
Urban	194 (47.55)	196 (39.36)	0.013
Rural	214 (52.45)	302 (60.64)	
Marital status, n (%)			
No	72 (17.65)	109 (21.89)	0.112
Yes	336 (82.35)	389 (78.11)	
Educational level, n (%)			
Below junior high school	162 (39.71)	270 (54.22)	< 0.001
Junior high school	88 (21.57)	117 (23.49)	
High school	96 (23.53)	64 (12.85)	
University degree or above	62 (15.20)	47 (9.44)	
Smoke, n (%)			
No	297 (72.79)	403 (80.92)	0.004
Yes	111 (27.21)	95 (19.08)	
Drink, n (%)			
No	273 (66.91)	403 (80.92)	< 0.001
Yes	135 (33.09)	95 (19.08)	
Sleep time duration, n (%)			
< 6	164 (40.20)	239 (47.99)	< 0.001
6–8	225 (55.15)	190 (38.15)	
> 8	19 (4.66)	69 (13.86)	
Social contact, n (%)			
No	202 (49.51)	309 (62.05)	< 0.001
Yes	206 (50.49)	189 (37.95)	
Fall, n (%)			
No	320 (78.43)	312 (62.65)	< 0.001
Yes	88 (21.57)	186 (37.35)	
Hip fracture, n (%)			
No	405 (99.26)	482 (96.79)	0.010
Yes	3 (0.74)	16 (3.21)	
Depression, n (%)			
No	263 (64.46)	191 (38.35)	< 0.001
Yes	145 (35.54)	307 (61.65)	
Pain, n (%)			
No	256 (62.75)	388 (77.91)	< 0.001
Yes	152 (37.25)	110 (22.09)	
Number of pains, n (%)			
0	158 (38.73)	115 (23.09)	< 0.001
1	46 (11.27)	39 (7.83)	
≥ 2	204 (50.00)	344 (69.08)	
Diabetes, n (%)			
No	297 (72.79)	344 (69.08)	0.221
Yes	111 (27.21)	154 (30.92)	
Hypertension, n (%)			
No	149 (36.52)	117 (23.49)	< 0.001
Yes	259 (63.48)	381 (76.51)	
Heart disease, n (%)			
No	258 (63.24)	272 (54.62)	0.009
Yes	150 (36.76)	226 (45.38)	
Mental disease, n (%)			
No	390 (95.59)	445 (89.36)	< 0.001
Yes	18 (4.41)	53 (10.64)	
Arthritis, n (%)			
No	203 (49.75)	171 (34.34)	< 0.001
Yes	205 (50.25)	327 (65.66)	
Dyslipidemia, n (%)			
No	215 (52.70)	246 (49.40)	0.323
Yes	193 (47.30)	252 (50.60)	
Kidney disease, n (%)			
No	336 (82.35)	371 (74.50)	0.004
Yes	72 (17.65)	127 (25.50)	
Lung disease, n (%)			
No	329 (80.64)	348 (69.88)	< 0.001
Yes	79 (19.36)	150 (30.12)	

ADL, activities of daily living; CES-D, Center for Epidemiologic Studies Depression Scale; M (Q₁, Q₃), median and interquartile range; SD, standard deviation

### Feature selection and model development

**Fig. 2:**
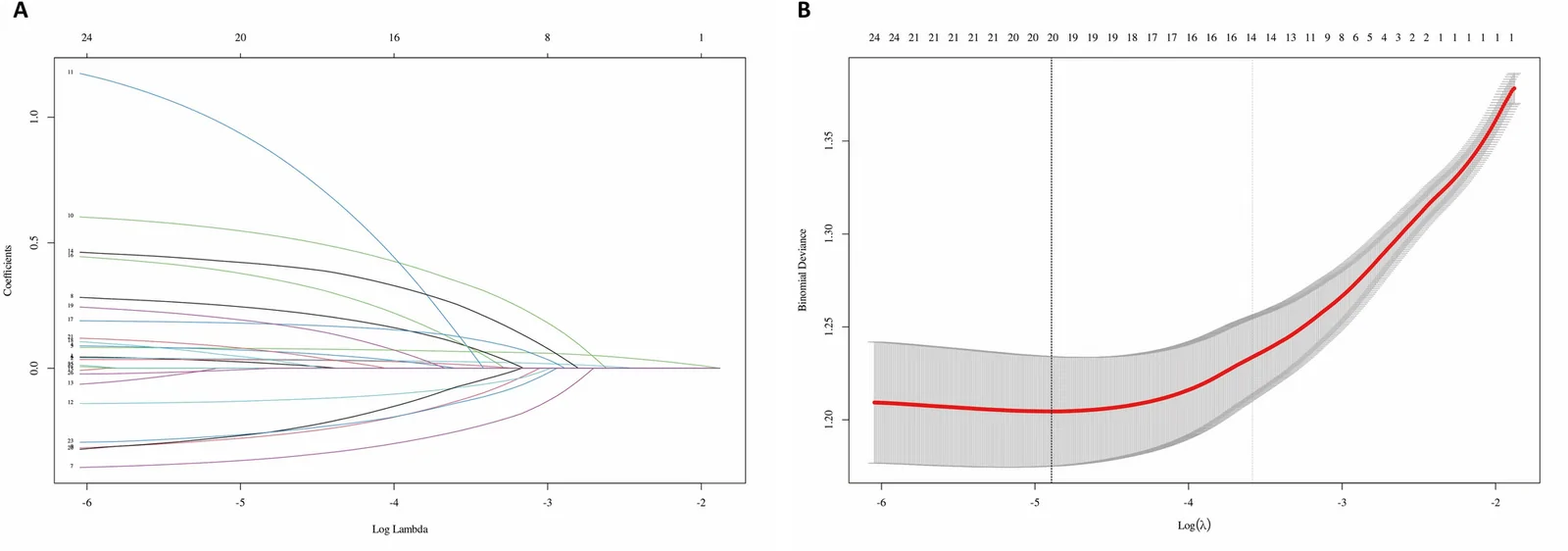
LASSO regression analysis. **A** Coefficient profiles of candidate variables with increasing penalty parameter (λ); **B** Selection of the optimal λ based on ten-fold cross-validation

**Fig. 3:**
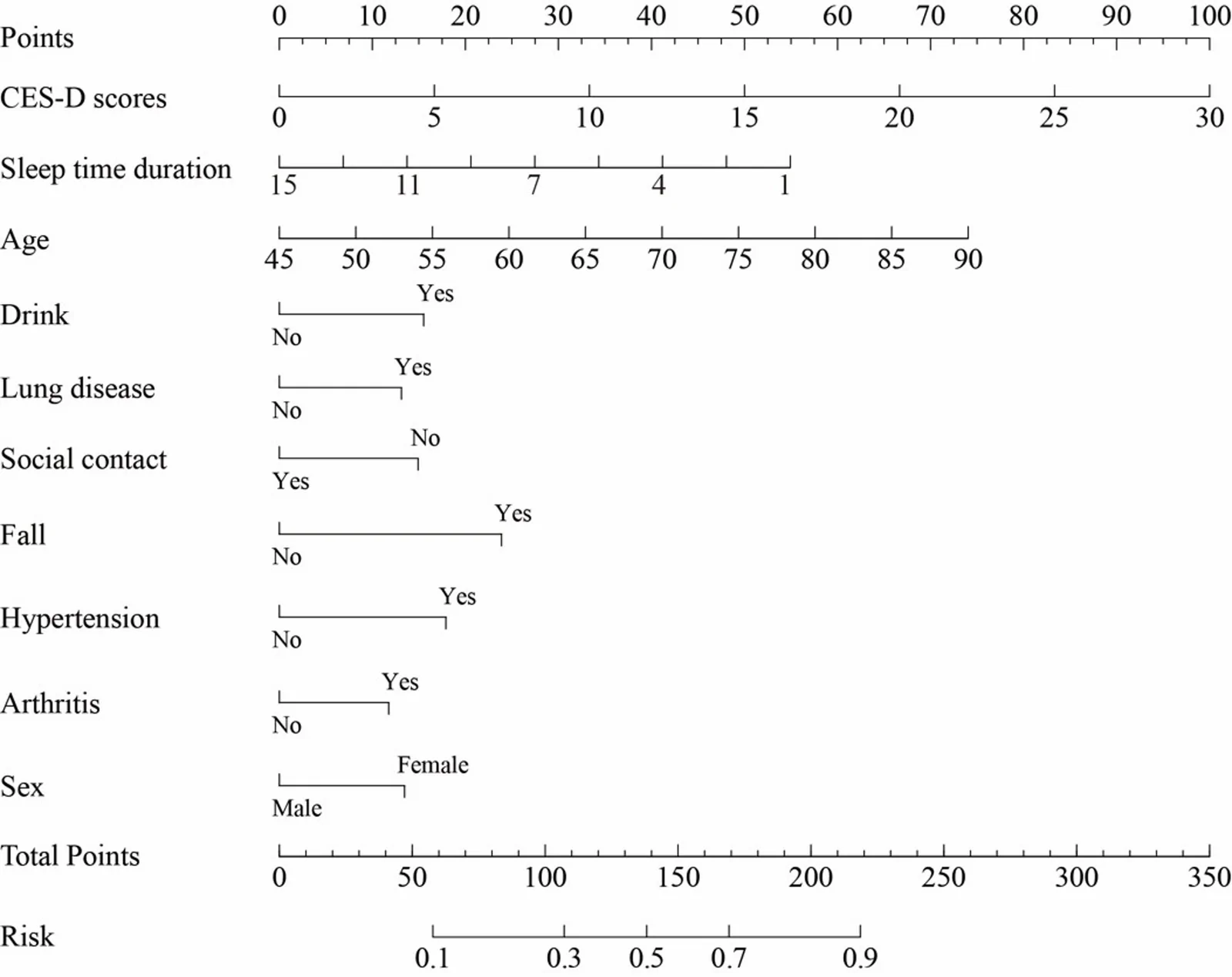
Nomogram for predicting the risk of ADL dysfunction among stroke patients based on selected predictors

To find important determinants of ADL dysfunction, LASSO regression analysis was applied to the training set. The coefficients of candidate variables progressively shrank toward zero with increasing penalization, and several variables were eliminated as the penalty parameter increased (Fig. 2A). Ten-fold cross-validation was used to determine the optimal value of the regularization parameter (λ), which was selected according to the minimum criterion (Fig. 2B). At this value, ten variables in all with non-zero coefficients were selected, including CES-D scores, sleep time duration, age, alcohol consumption, lung disease, social contact, falls, hypertension, arthritis, and sex. A multivariable logistic regression model was then created using these factors. All of the selected indicators were kept as independent predictors of ADL dysfunction since they were still statistically significant (*P* < 0.05) (Table S2). To determine individualized risk of ADL dysfunction, a nomogram combining these variables was created (Fig. 3).

### Model interpretation

**Fig. 4:**
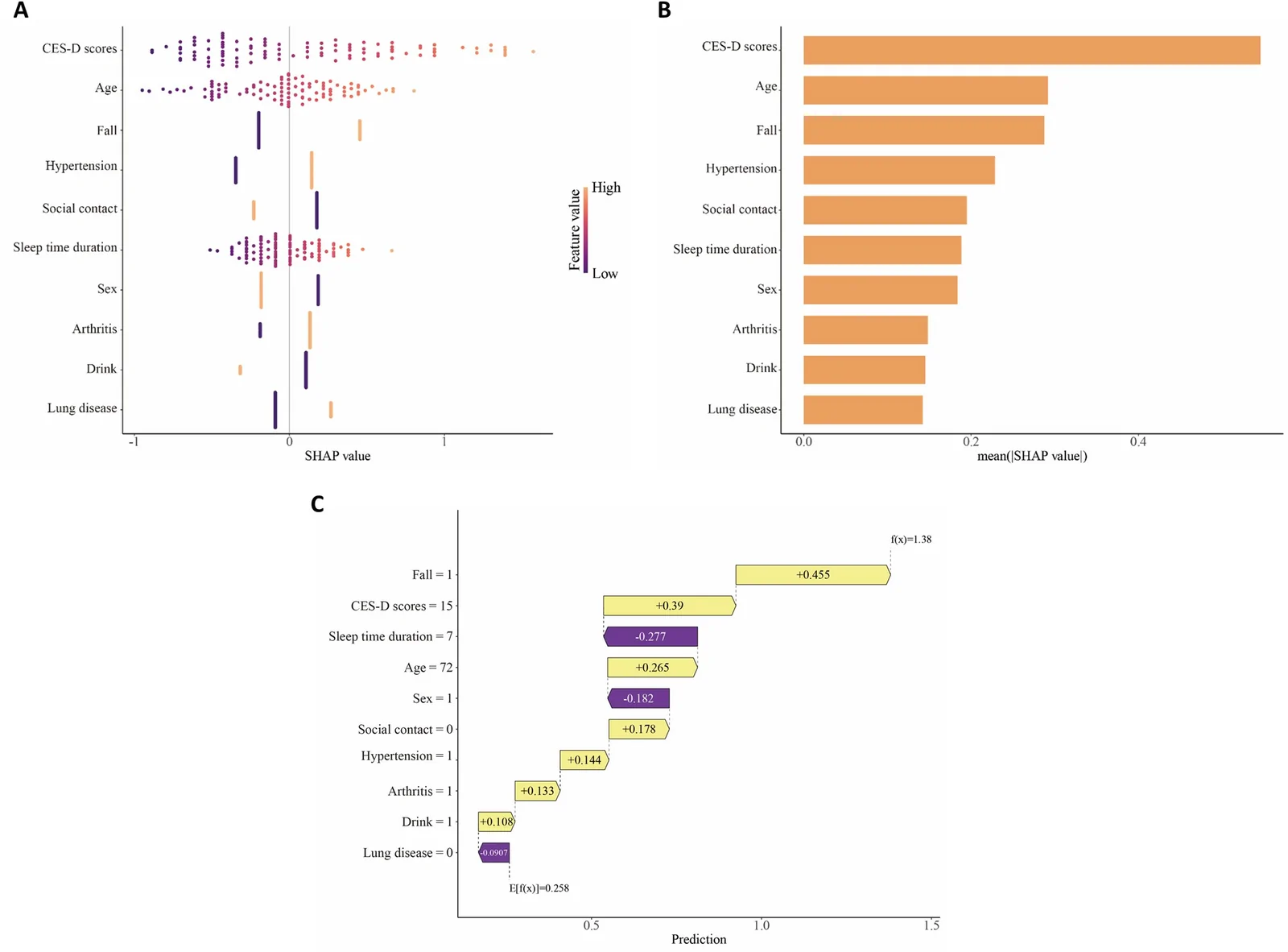
SHAP interpretation of the predictive model. **A** SHAP summary plot of the direction and magnitude of predictor effects; **B** Variable importance ranked by mean absolute SHAP values; **C** SHAP waterfall plot of individual-level prediction. Sex (0 = female, 1 = male); Other categorical variables (0 = no, 1 = yes). Age, CES-D scores, and sleep time duration were analyzed as continuous variables

The contribution of each predictor to ADL dysfunction was measured using SHAP analysis. Higher CES-D scores and older age generally contributed to increased model predictions, while the distribution of SHAP values indicated variability in the magnitude of their effects across individuals. Falls, hypertension, lack of social contact, and arthritis also contributed positively to the prediction, whereas sleep time duration, sex, drinking, and lung disease exhibited negative contributions in certain ranges (Fig. 4A). According to the mean absolute SHAP values, the CES-D score emerged as the most significant predictor of the model’s output. Following this, age, falls, hypertension, and social contact were identified as the subsequent key contributors to variable importance. Sleep time duration and sex showed moderate influence, whereas arthritis, drinking, and lung disease contributed relatively less (Fig. 4B). At the individual level, the SHAP waterfall plot illustrated the cumulative effect of predictors on model output. Increased risk was primarily driven by falls, elevated CES-D scores, advanced age, absence of social contact, hypertension, arthritis and drinking status, while longer sleep time duration, male sex, and absence of lung disease contributed to risk reduction (Fig. 4C).

### Model performance and validation

**Fig. 5:**
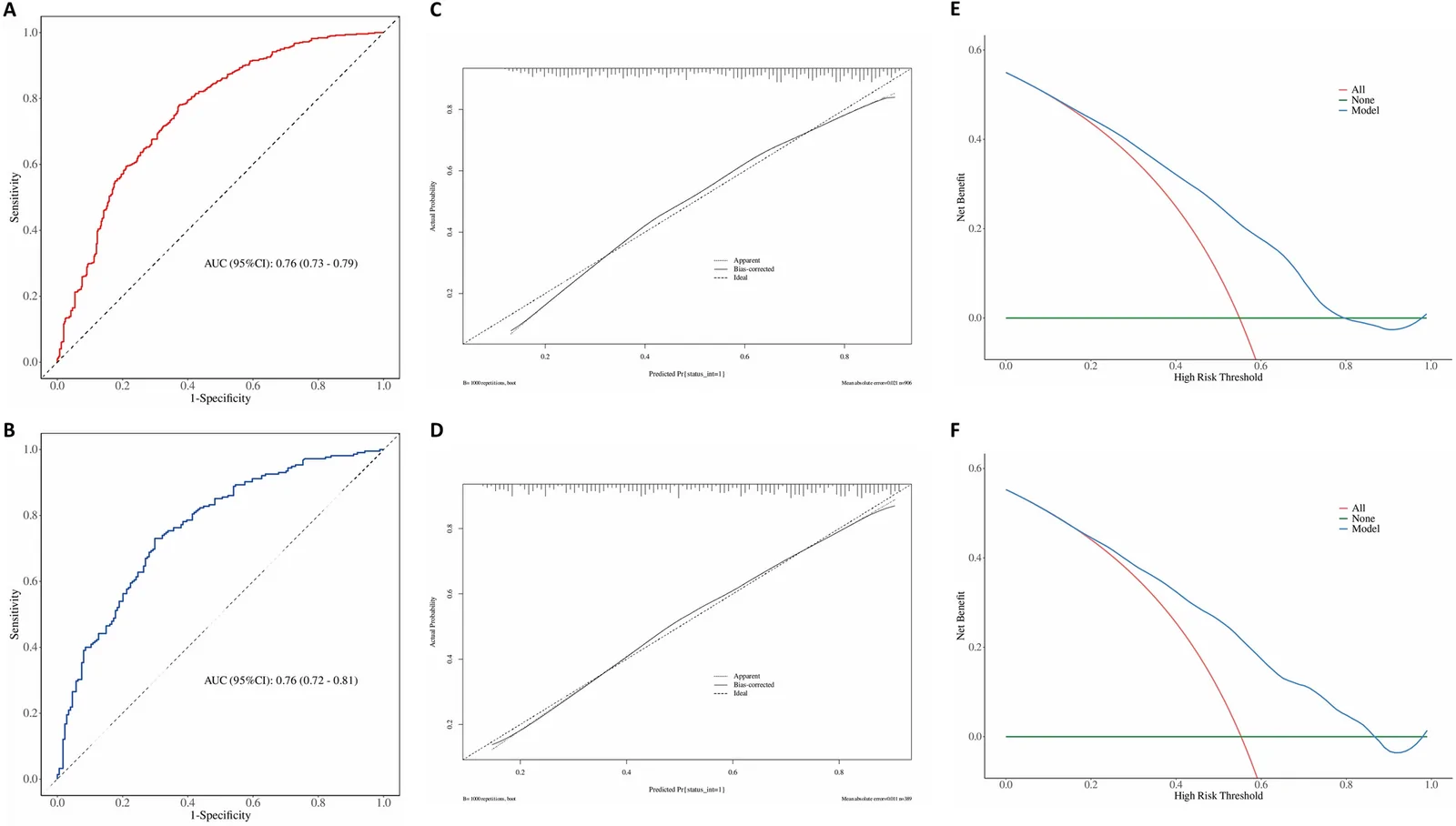
Model performance and validation. **A**, **B** Receiver operating characteristic (ROC) curves in the training and validation sets; **C**, **D** Calibration curves in the training and validation sets; **E**, **F** Decision curve analysis (DCA) in the training and validation sets

In both the training and validation sets, the model’s prediction performance was thoroughly assessed. In the training set, the model demonstrated acceptable discriminative ability, with an AUC of 0.76 (95% CI: 0.73–0.79). Consistent results were observed in the validation set (AUC: 0.76; 95% CI: 0.72–0.81), supporting the stability of the model in internal validation (Fig. 5A–B). Sensitivity analysis using complete-case data yielded similar results, with an AUC of 0.80 (95% CI: 0.78–0.83), supporting the stability of the model (Figure S1). In addition, 10-fold cross-validation demonstrated consistent discriminative performance, yielding an AUC of 0.748 (95% CI: 0.721–0.775) (Figure S2), further supporting the robustness of the model. Calibration curves demonstrated good agreement between predicted and observed outcomes in both the training and validation sets (Fig. 5C–D). The Hosmer–Lemeshow goodness-of-fit test indicated satisfactory calibration in the training set (χ² = 11.81, df = 8, *P* = 0.16) and validation set (χ² = 1.45, df = 8, *P* = 0.99). Furthermore, DCA demonstrated that the model yielded higher net benefit than the treat-all and treat-none strategies across threshold probabilities of approximately 0.20–0.80 in both the training and validation sets, supporting its potential clinical utility (Fig. 5E–F).

## Discussion

In this study, a prediction model for ADL dysfunction among stroke survivors was developed and validated using data from CHARLS. The final model incorporated ten predictors, including CES-D scores, age, sleep time duration, drinking, lung disease, social contact, falls, hypertension, arthritis, and sex. The model demonstrated acceptable discrimination, calibration, and potential clinical utility; however, external validation and prospective studies are required before its application in routine clinical practice. By incorporating demographic, clinical, lifestyle, and psychosocial variables, the model integrates multiple domains associated with ADL dysfunction and may assist in identifying stroke survivors with functional impairment.

Among the identified predictors, depressive symptoms (CES-D scores) emerged as the most influential factor. The strong association between depression and ADL dysfunction is consistent with prior studies, which have indicated that depressive symptoms often co-occurring with functional impairments and serving as a reliable predictor of subsequent ADL impairment [39, 40]. A UK national survey revealed ADL disability and depression are strongly correlated, with a potential cumulative impact [41]. Moreover, the reciprocal association between depressed symptoms and ADL disability has been extensively investigated in later cohort studies [42, 43]. However, due to the cross-sectional design, causality and temporal relationships between depression and ADL dysfunction cannot be established. Depressive symptoms are highly prevalent among stroke survivors, affecting at least one-third of patients, and may further complicate management beyond physical disabilities alone [44]. The pathophysiology of post-stroke depression involves complex interactions among several biological pathways, such as abnormal glutamatergic signaling, dysregulation of monoaminergic neurotransmission, disruptions in gut microbiota-brain communication, neuroinflammation mediated by the hypothalamic-pituitary-adrenal (HPA) axis, and decreased expression of brain-derived neurotrophic factor [45]. Although the exact mechanisms linking depression and ADL dysfunction remain to be fully elucidated, the consistent association observed across studies indicates the importance of incorporating depressive symptom assessment into functional evaluation. Targeting depression may therefore represent a critical component of comprehensive rehabilitation strategies aimed at improving ADL outcomes in stroke survivors.

Demographic factors also played an important role in ADL dysfunction. Increasing age was strongly associated with higher risk, which is in line with earlier data showing that older stroke survivors tend to have poorer functional recovery due to reduced physiological reserve and an increased comorbidity burden [46]. One of the hallmarks of biological aging is chronic low-grade systemic inflammation, which contributes to muscle catabolism, frailty, and functional deterioration [47]. Sex differences were observed, with female stroke survivors showing a higher risk of ADL dysfunction. Women are more likely to have comorbidities and pre-existing disabilities at the time of stroke onset, which may adversely affect recovery [48]. In addition, the loss of estrogen’s neuroprotective effects after menopause may also contribute to these disparities, as estrogen has been shown to regulate inflammatory responses and promote neuronal survival following ischemic injury [49]. These findings emphasize the need to consider sociodemographic context when assessing rehabilitation needs and planning individualized interventions.

In terms of lifestyle factors, sleep time duration showed a significant relationship with functional status, suggesting that insufficient sleep may adversely affect ADL. Sleep disturbances are common after stroke and have been linked to depressive symptoms, cognitive dysfunction, and impaired neuroplasticity [50]. Chronic short sleep increases sympathetic nervous system activity and elevates cortisol levels. This systemic stress response can lead to poorly controlled hypertension and insulin resistance, both of which impair small vessel integrity and delay functional healing [51]. Drinking was also retained in the model. Previous studies have suggested that the relationship between alcohol consumption and stroke-related outcomes is complex, with some reports describing J-shaped associations according to the level of alcohol intake [52, 53]. Nevertheless, excessive alcohol exposure and its metabolite acetaldehyde may inhibit brain-derived neurotrophic factor expression and disrupt hippocampal and cortical long-term potentiation, impairing neuroplasticity and limiting functional recovery in ADL [54]. Given that CHARLS only recorded drinking status rather than the amount or pattern of alcohol consumption, the observed association should be interpreted cautiously. In addition, reduced social contact was identified as a significant predictor, highlighting the importance of social support in post-stroke recovery. Social contact serves as a critical buffer against post-stroke depression and anxiety, which are known to impede physical rehabilitation. According to the Stress-Buffering Hypothesis, robust social support modulates the HPA axis, lowering cortisol levels and systemic inflammation, thereby creating a more favorable physiological environment for brain repair [55, 56].

Several clinical factors were also significantly associated with ADL dysfunction, including falls, hypertension, arthritis, and lung disease. Falls may reflect underlying impairments in balance, mobility, and neuromuscular control, and are both a marker and contributor to functional decline. Hypertension, as a major vascular risk factor, may exacerbate cerebral damage and hinder recovery through ongoing vascular dysfunction [57]. Arthritis can further limit mobility and physical function due to pain and joint stiffness, thereby compounding disability after stroke [58]. In addition, lung disease may impair exercise tolerance and oxygen delivery, reducing overall physical capacity and participation in rehabilitation [59]. These conditions may interact to further restrict ADL independence.

The identified predictors have important implications for rehabilitation planning. Several factors retained in the model, including depressive symptoms, social contact, sleep duration, drinking status, falls, and hypertension, are potentially modifiable and may represent targets for intervention aimed at preserving or improving functional independence. In contrast, non-modifiable factors such as age and sex may assist in identifying stroke survivors at greater risk of ADL dysfunction who could benefit from closer monitoring and individualized rehabilitation strategies. Together, these findings support a multidimensional approach to post-stroke functional assessment that incorporates demographic, clinical, lifestyle, and psychosocial factors.

## Conclusions

A prediction model for ADL dysfunction among stroke survivors was developed and internally validated using a large, nationally representative dataset. The model showed acceptable discriminative ability, good calibration, and potential clinical utility. By facilitating the identification of individuals with ADL dysfunction, this tool may support more targeted rehabilitation strategies and contribute to improved functional outcomes in stroke survivors. Further external validation and prospective studies are needed to confirm its generalizability and clinical value.

## Limitations

First, cross-sectional data from the CHARLS 2018 wave served as the basis for this analysis, which restricts the capacity to determine causal linkages or temporal relationships between predictors and ADL dysfunction. Reverse causation cannot be excluded; for example, ADL dysfunction may contribute to depressive symptoms, rather than depression solely contributing to functional impairment. Second, all of the variables were from self-reported data, which could lead to misclassification and recall bias. Third, some potentially important variables, such as stroke severity, lesion characteristics, and detailed rehabilitation interventions, were not available in the dataset, which may have affected model performance. Because stroke severity is a well-established predictor of post-stroke functional outcomes, its absence may have reduced the predictive accuracy of the model [60]. Fourth, the model was developed and validated using a split-sample internal validation approach within the same dataset; therefore, its generalizability should be confirmed by external validation in separate cohorts. Lastly, the results may not be as applicable to other populations or healthcare settings because the study cohort was limited to middle-aged and older persons in China.

Longitudinal data should be included in future studies to assess the model’s predictive accuracy over time and to more accurately reflect changes in functional status following a stroke. In addition, integrating clinical indicators such as stroke severity, imaging findings, and rehabilitation treatment details may further improve model accuracy and enhance its applicability in clinical practice. Further investigation into the underlying biological mechanisms linking these predictors to ADL dysfunction may also provide deeper insights into post-stroke functional impairment.

## Supplementary Information

Supplementary Material 1. Figure S1. Receiver operating characteristic (ROC) curve for the sensitivity analysis using complete-case data

Supplementary Material 2. Figure S2. Receiver operating characteristic curve from 10-fold cross-validation.

Supplementary Material 3.

## Data Availability

No datasets were generated or analysed during the current study.

## Abbreviations

ADL: Activities of daily living
AUC: Area under the receiver operating characteristic curve
BADL: Basic activities of daily living
CES-D: Center for Epidemiologic Studies Depression Scale
CHARLS: China Health and Retirement Longitudinal Study
CIs: Confidence intervals
DCA: Decision curve analysis
EPV: events-per-variable
HPA: Hypothalamic-pituitary-adrenal
IADL: Instrumental activities of daily living
IQR: interquartile ranges
LASSO: Least absolute shrinkage and selection operator
ORs: Odds ratios
ROC: Receiver operating characteristic
SD: Standard deviation
SHAP: SHapley Additive exPlanations
TRIPOD: Transparent Reporting of a multivariable prediction model for Individual Prognosis Or Diagnosis
