# Supplementary material for: Development and validation of a prediction model for activities of daily living dysfunction among stroke survivors: insights from the CHARLS cohort
Source: J Neuroeng Rehabil. 2026 Jun 21;23:258. doi: 10.1186/s12984-026-02063-x (PMC13548701; doi:10.1186/s12984-026-02063-x)
Supplement: Supplementary file 1 [file 12984_2026_2063_MOESM3_ESM.docx]

Table S1 Distribution of variables with missing data in cross-sectional study

| **Variables** | **Total** | **Freq** | **Miss (Percent)** |
| --- | --- | --- | --- |
| BADL | 1,549 | 1,544 | 5 (0.32) |
| IADL | 1,549 | 1,544 | 5 (0.32) |
| Social contact | 1,549 | 1,546 | 3 (0.19) |
| Number of healthy children | 1,549 | 1,436 | 113 (7.30) |
| CES-D scores | 1,549 | 1,295 | 254 (16.40) |
| Fall | 1,549 | 1,546 | 3 (0.19) |
| Hip fracture | 1,549 | 1,546 | 3 (0.19) |
| Pain | 1,549 | 1,546 | 3 (0.19) |
| Sleep time duration | 1,549 | 1,546 | 3 (0.19) |
| Daytime napping | 1,549 | 1,546 | 3 (0.19) |
| Smoke | 1,549 | 1,546 | 3 (0.19) |
| Drink | 1,549 | 1,546 | 3 (0.19) |

CES-D: Center for Epidemiologic Studies Depression Scale; BADL: basic activities of daily living; IADL: instrumental activities of daily living

Table S2 Multivariate logistic regression analysis of the training set

| **Variables** | **β** | **SE** | **Z** | **P** | **Odds ratio (95% CI)** |
| --- | --- | --- | --- | --- | --- |
| CES-D scores | 0.09 | 0.01 | 7.43 | < 0.001 | 1.10 (1.07–1.12) |
| Sleep time duration | 0.09 | 0.04 | 2.64 | 0.008 | 1.10 (1.02–1.18) |
| Age | 0.04 | 0.01 | 5.11 | < 0.001 | 1.05 (1.03–1.06) |
| Drink |  |  |  |  |  |
| Yes |  |  |  |  | 1.00 (Reference) |
| No | -0.42 | 0.18 | -2.34 | 0.019 | 0.65 (0.46–0.93) |
| Lung disease |  |  |  |  |  |
| No |  |  |  |  | 1.00 (Reference) |
| Yes | 0.36 | 0.18 | 2.02 | 0.044 | 1.43 (1.01–2.03) |
| Social contact |  |  |  |  |  |
| No |  |  |  |  | 1.00 (Reference) |
| Yes | -0.41 | 0.15 | -2.69 | 0.007 | 0.67 (0.49–0.90) |
| Fall |  |  |  |  |  |
| No |  |  |  |  | 1.00 (Reference) |
| Yes | 0.65 | 0.17 | 3.89 | < 0.001 | 1.92 (1.38–2.67) |
| Hypertension |  |  |  |  |  |
| No |  |  |  |  | 1.00 (Reference) |
| Yes | 0.49 | 0.16 | 2.98 | 0.003 | 1.63 (1.18–2.25) |
| Arthritis |  |  |  |  |  |
| No |  |  |  |  | 1.00 (Reference) |
| Yes | 0.32 | 0.16 | 2.06 | 0.040 | 1.38 (1.02–1.87) |
| Sex |  |  |  |  |  |
| Female |  |  |  |  | 1.00 (Reference) |
| Male | -0.37 | 0.16 | -2.27 | 0.023 | 0.69 (0.50–0.95) |
| Intercept | -4.60 | 0.68 | -6.72 | < 0.001 | 0.01 (0.00–0.04) |

**Note:** β, regression coefficient; SE, standard error; CI, confidence interval. The complete model specification is provided in this table. The predicted probability of ADL dysfunction was calculated as $P=1/[1+\exp(-\text{logit}(P))]$, where $\text{logit}(P)=\beta_{0}+\sum\beta_{i}X_{i}$. The intercept ($\beta_{0}$) was -4.60.

Table S3 Comparison of baseline characteristics between the training and validation sets in stroke patients

| **Variables** | **Training set (n = 906)** | **Validation set (n = 389)** | **P** |
| --- | --- | --- | --- |
| Age, Mean ± SD | 66.11 ± 8.77 | 65.88 ± 9.32 | 0.672 |
| Family size, M (Q₁, Q₃) | 2.00 (2.00, 3.00) | 2.00 (2.00, 3.00) | 0.203 |
| Number of healthy children,  M (Q₁, Q₃) | 3.00 (2.00, 4.00) | 2.00 (2.00, 4.00) | 0.216 |
| CES-D scores, M (Q₁, Q₃) | 9.00 (5.00, 16.00) | 10.00 (6.00, 15.00) | 0.747 |
| Sleep time duration, M (Q₁, Q₃) | 6.00 (4.00, 7.50) | 6.00 (4.00, 8.00) | 0.464 |
| Daytime napping, M (Q₁, Q₃) | 30.00 (0.00, 60.00) | 30.00 (0.00, 60.00) | 0.592 |
| Age, n (%) |  |  | 0.226 |
| < 65 | 377 (41.61) | 176 (45.24) |  |
| ≥ 65 | 529 (58.39) | 213 (54.76) |  |
| Sex, n (%) |  |  | 0.119 |
| Female | 448 (49.45) | 174 (44.73) |  |
| Male | 458 (50.55) | 215 (55.27) |  |
| Residence, n (%) |  |  | 0.580 |
| Urban | 390 (43.05) | 161 (41.39) |  |
| Rural | 516 (56.95) | 228 (58.61) |  |
| Marital status, n (%) |  |  | 0.692 |
| No | 181 (19.98) | 74 (19.02) |  |
| Yes | 725 (80.02) | 315 (80.98) |  |
| Educational level, n (%) |  |  | 0.779 |
| Below junior high school | 432 (47.68) | 187 (48.07) |  |
| Junior high school | 205 (22.63) | 79 (20.31) |  |
| High school | 160 (17.66) | 75 (19.28) |  |
| University degree or above | 109 (12.03) | 48 (12.34) |  |
| Smoke, n (%) |  |  | 0.100 |
| No | 700 (77.26) | 284 (73.01) |  |
| Yes | 206 (22.74) | 105 (26.99) |  |
| Drink, n (%) |  |  | 0.171 |
| No | 676 (74.61) | 276 (70.95) |  |
| Yes | 230 (25.39) | 113 (29.05) |  |
| Sleep time duration, n (%) |  |  | 0.113 |
| < 6 | 403 (44.48) | 151 (38.82) |  |
| 6–8 | 415 (45.81) | 190 (48.84) |  |
| > 8 | 88 (9.71) | 48 (12.34) |  |
| Social contact, n (%) |  |  | 0.694 |
| No | 511 (56.40) | 224 (57.58) |  |
| Yes | 395 (43.60) | 165 (42.42) |  |
| Fall, n (%) |  |  | 0.667 |
| No | 632 (69.76) | 276 (70.95) |  |
| Yes | 274 (30.24) | 113 (29.05) |  |
| Hip fracture, n (%) |  |  | 0.321 |
| No | 887 (97.90) | 384 (98.71) |  |
| Yes | 19 (2.10) | 5 (1.29) |  |
| Depression, n (%) |  |  | 0.354 |
| No | 454 (50.11) | 184 (47.30) |  |
| Yes | 452 (49.89) | 205 (52.70) |  |
| Pain, n (%) |  |  | 0.815 |
| No | 644 (71.08) | 279 (71.72) |  |
| Yes | 262 (28.92) | 110 (28.28) |  |
| Number of pains, n (%) |  |  | 0.717 |
| 0 | 273 (30.13) | 119 (30.59) |  |
| 1 | 85 (9.38) | 31 (7.97) |  |
| ≥ 2 | 548 (60.49) | 239 (61.44) |  |
| Diabetes, n (%) |  |  | 0.588 |
| No | 641 (70.75) | 281 (72.24) |  |
| Yes | 265 (29.25) | 108 (27.76) |  |
| Hypertension, n (%) |  |  | 0.984 |
| No | 266 (29.36) | 114 (29.31) |  |
| Yes | 640 (70.64) | 275 (70.69) |  |
| Heart disease, n (%) |  |  | 0.695 |
| No | 530 (58.50) | 223 (57.33) |  |
| Yes | 376 (41.50) | 166 (42.67) |  |
| Mental disease, n (%) |  |  | 0.814 |
| No | 835 (92.16) | 360 (92.54) |  |
| Yes | 71 (7.84) | 29 (7.46) |  |
| Arthritis, n (%) |  |  | 0.903 |
| No | 374 (41.28) | 162 (41.65) |  |
| Yes | 532 (58.72) | 227 (58.35) |  |
| Dyslipidemia, n (%) |  |  | 0.928 |
| No | 461 (50.88) | 199 (51.16) |  |
| Yes | 445 (49.12) | 190 (48.84) |  |
| Kidney disease, n (%) |  |  | 0.575 |
| No | 707 (78.04) | 309 (79.43) |  |
| Yes | 199 (21.96) | 80 (20.57) |  |
| Lung disease, n (%) |  |  | 0.650 |
| No | 677 (74.72) | 286 (73.52) |  |
| Yes | 229 (25.28) | 103 (26.48) |  |
| BADL dysfunction, n (%) |  |  | 0.534 |
| No | 557 (61.48) | 232 (59.64) |  |
| Yes | 349 (38.52) | 157 (40.36) |  |
| IADL dysfunction, n (%) |  |  | 0.614 |
| No | 468 (51.66) | 195 (50.13) |  |
| Yes | 438 (48.34) | 194 (49.87) |  |
| ADL dysfunction, n (%) |  |  | 0.920 |
| No | 408 (45.03) | 174 (44.73) |  |
| Yes | 498 (54.97) | 215 (55.27) |  |
